# Prostogrit mitigates testosterone/estradiol induced prostatic enlargement/remodeling in rat model of benign prostatic hyperplasia and fine-tunes prostatic expression of *Adra1a* and *Il6* genes

**DOI:** 10.3389/fendo.2025.1663917

**Published:** 2025-12-15

**Authors:** Acharya Balkrishna, Sandeep Sinha, Venu Pamidiboina, Meenu Tomer, Rani Singh, Rishabh Dev, Anurag Varshney

**Affiliations:** 1 Drug Discovery and Development Division, Patanjali Research Foundation, Haridwar, India; 2 Department of Allied and Applied Sciences, University of Patanjali, Haridwar, India; 3 Patanjali UK Trust, Glasgow, United Kingdom

**Keywords:** Ayurveda, benign prostate hyperplasia, estradiol, Prostogrit, testosterone, α1A adrenoceptor

## Abstract

**Background:**

Benign prostate hyperplasia (BPH), the pathological basis of benign prostatic syndrome (BPS), is an ageing-associated, androgen-driven, non-malignant growth of the prostate, with an inflammatory etiology. It is characterized by hyperproliferation of prostate cells and bothersome lower urinary tract symptoms. Current therapies for BPH provide both symptomatic relief and are disease modifying. However, they are associated with treatment limiting adverse effects. Accordingly, safer and effective alternatives to conventional treatments are required. Prostogrit is a novel, Ayurvedic medicine, indicated for the treatment of BPH. The objective of the current study was to evaluate the pharmacological effects of Prostogrit in rat model of BPH.

**Methods:**

Ultra high performance liquid chromatography was employed to detect and quantify the phytocompounds present in Prostogrit. BPH was induced in male Sprague Dawley (SD) rats by subcutaneous administration of testosterone propionate (TP) + estradiol benzoate (EB). Rats were administered Prostogrit at the doses of 10, 30, 100 and 300 mg/kg, twice daily (*b.i.d.)*. Finasteride administered once daily *(q.d.)* at the dose of 1 mg/kg was employed as the method control. The experimental readouts included measurements of the weights of prostate, seminal vesicles and urinary bladder; histological evaluation of prostatic tissue along with mRNA expression of alpha 1A adrenoceptor (*Adra1a*) and interleukin 6 (*Il6*) genes.

**Results:**

Phytochemical analysis of Prostogrit revealed the presence of phytometabolites, namely guggulsterone, gallic acid, 5-hydroxymethylfurfural (5-HMF), methyl gallate, cinnamic acid and piperine. In the *in vivo* experiment, Prostogrit restored the TP+EB-induced increase in the relative weights of prostate and urinary bladder. It ameliorated the TP+EB-evoked epithelial proliferation, acinar hypertrophy and infiltration of inflammatory cells in the prostate stroma, in a dose-dependent fashion. Additionally, Prostogrit could also suppress the TP+EB-induced increase in the mRNA expression of *Adra1a* and *Il6* genes in prostatic tissue.

**Conclusion:**

The findings of the current proof of concept study suggest that Prostogrit has pharmacological effects in an animal model of BPH. Accordingly, it possesses preclinical potential for the pharmacotherapeutic management of BPH, which merits further investigations.

## 1 Introduction

Benign prostate hyperplasia, the pathological substratum of clinical benign prostatic syndrome, is a non-cancerous enlargement of the prostate gland that arises from hormonal influences and the natural ageing process. It adversely impacts the quality of life of patients due to urinary bladder outlet obstruction and the consequent lower urinary tract symptoms (LUTS) (1, 2). The development and growth of prostate gland mainly depends on androgen stimulation by dihydrotestosterone (DHT), an active metabolite of testosterone. Bio-conversion of testosterone to DHT in prostate gland is catalyzed by the Type II 5α-reductase enzyme (3). Although, serum testosterone levels decline with age, the dihydrotestosterone (DHT) levels in prostate gland has been contrastingly reported to be at a normal level (4). Binding of DHT to the androgen receptor (AR) has been reported to cause prostatic enlargement through diverse pathophysiological mechanisms (5, 6). Additionally, patients with benign prostatic hyperplasia (BPH) have been reported to have higher serum and prostatic levels of estradiol as well. Estradiol, produced from testosterone via aromatase enzyme, is known to potentiate pathological growth and inflammation of the prostate gland (7). In addition, chronic inflammation within the prostate gland is closely related with the development of BPH, reflected by an elevated number of leukocytes in the epithelial and stromal sections. The invading inflammatory cell population have been known to trigger tissue damage via release of inflammatory mediators (8). Histological changes observed in BPS patients have been reported to result in appearance of distinct prostatic nodules, inflammation, fibrosis, and alterations in smooth muscle activity, which can cause partial or complete urethral obstruction (9, 10).

Standard drugs used clinically to treat BPS associated with BPH include 5α-reductase inhibitors (5ARIs) like finasteride and dutasteride and α1-adrenoceptor antagonists (like silodosin and tamsulosin). 5α-reductase inhibitors are known to prevent the conversion of testosterone to DHT in the prostate gland, thereby reducing its intra-prostatic levels (11). α_1_-adrenoceptor antagonists on the other hand act by relaxing the smooth muscle of prostate gland, which thereby relieves urethral obstruction (12). Both medications have been reported to demonstrate effectiveness in treating BPS. However, it is important to note that they are associated with several treatment limiting adverse effects (13). Hence, there is an increasing demand for alternative medicines that on one hand minimize side effects and on the other enhance patient well-being. In this light, from a therapeutic perspective, botanical drug-based formulations are becoming increasingly popularized (14).

Prostogrit is an Ayurvedic medicine that contains botanical drugs and minerals. It has been specifically developed for the treatment of BPS associated with BPH. Our study aimed to assess its *in vivo* pharmacological effects in a rat model of BPH induced by a combination of testosterone propionate (TP) and estradiol benzoate (EB). TP+EB-induced BPH model in rats has been utilized for evaluating the pharmacological effectiveness of therapeutic modalities against BPS, as this model leads to development of a BPH-like disease pathology (15, 16). In the current study, Prostogrit was orally administered to Sprague Dawley rats, 14-days prior to disease induction. This was followed by administration of TP+EB at the ratio of 100:1, subcutaneously for 35-consecutive days. At study termination, the animals were humanely sacrificed and prostate glands, seminal vesicles and urinary bladder were excised and weighed. Then their relative organ weights with respect to the animal’s terminal body weights were computed. Ventral lobes of the prostate were subjected to histological and mRNA expression analysis. Finasteride, a 5α-reductase inhibitor was used as the reference control to validate the model. Additionally, the phytochemical analysis of Prostogrit was also conducted to detect and quantify the bioactive phytometabolites, with an aim of explaining the outcomes of the *in vivo* experiment.

## 2 Materials and methods

### 2.1 Test article, chemicals and reagents

Prostogrit (Internal Batch No: PRF/CHI/0823/0858) was sourced from Divya Pharmacy, Haridwar, India. The individual botanical drugs and minerals present in Prostogrit are detailed in **Table 1**. Finasteride tablets (Curlzfin-1 mg; Make: Canixa Life Sciences Pvt. Ltd., India) was purchased from the local market. Additionally, Testosterone Propionate (Cat. # T0028), Estradiol Benzoate (Cat. # E0329), gallic acid (Cat. # G0011), 5-HMF (Cat. # H0269), and methyl gallate (Cat. # G0017) were obtained from Tokyo Chemical Industry (India) Pvt. Ltd. Thiopentone was sourced from Neon Laboratories Limited, India. Benzoic acid (Cat. # HY-N0216) was purchased from MCE, USA, and cinnamic acid (Cat. # 29955) was obtained from Sisco Research Laboratories Pvt. Ltd., India. Guggulsterone E (Cat. # G009) and guggulsterone Z (Cat. # G010) were procured from Natural Remedies, India, while piperine (Cat. # P49007) was sourced from Sigma Aldrich, USA. Additional materials included the Verso cDNA synthesis kit (Cat. # AB-1453/B) and Trizol reagent (Cat. # 15596018) from ThermoFisher Scientific, USA and the PowerUp SYBR Green Master Mix (Cat. #A25742) from Applied Biosystems, USA. HBSS (Cat. # TS1098) was obtained from Himedia, India, whereas methylcellulose (Cat. # 04635) was bought from Loba Chemie Pvt. Ltd. (India) and RNAProtect tissue reagent (Cat. # 76104) was purchased from Qiagen, Germany.

**Table 1:** Composition of prostogrit.

Sl. no	Scientific name	Traditional name	Sanskrit binomial name	Part of the plant	Quantity (mg)
Extracts of:					
1	*Acorus calamus L.*	Vacha	उग्रगन्धक: वच: (Ugragandhakaḥ vacaḥ)	Rhizome	5
2	*Cyperus rotundus L.*	Motha	जलमुक् मुस्तक: (Jalamuk mustakaḥ)	Rhizome	5
3	*Cedrus deodara (Roxb. ex. D.Don) G.Don*	Devdaru	देवद्रुज: दृढकाष ्ठ : (Devadrujaḥ dṛḍhakāṣṭhaḥ)	Hardwood	5
4	*Cinnamomum zeylanicum Blume*	Dalchini	सुगन्धि जातकम् त्वक् (Sugandhijātakam tvak)	Bark	5
5	*Cinnamomum tamala (Buch. -Ham.) T.Nees & C.H.Eberm.*	Tejpatra	गन्धि जातकम् तमालम् (Gandhijātakam tamālam)	Leaf	5
6	*Curcuma longa L.*	Haldi	ह रि द्रका प ीतरागा (Haridrakā pītarāgā)	Rhizome	5
7	*Aconitum heterophyllum Wall.*	Kadvi atis	वत्सनाभि का घ ुण ेष ्टा (Vatsanābhikā ghuṇeṣṭā)	Root	5
8	*Berberis aristata DC.*	Daruhaldi	प ीतद्रुक: हरि द्रु: (Pītadrukaḥ haridruḥ)	Stem/Root	5
9	*Plumbago zeylanica L.*	Chitrak	चि त्रक: सि तसुम: (Citrakaḥ sitasumaḥ)	Root Bark	5
10	*Operculina turpethum (L.) Silva Manso*	Nishoth	प ीनश ल्कम् ताम्रमुकुलम् (Pīnaśalkam tāmramukulam)	Root	5
11	*Baliospermum montanum Müll.Arg.*	Danti	दन्ति का अरोमपत्रा (Dantikā aromapatrā)	Root	5
12	*Piper longum L.*	Pipplamool	कणि काप ि प ्पली (Kaṇikā pippalī)	Root	5
13	*Elettaria cardamomum* var. *major Baker*	Elaichi	एलि का झ ि रि प ्रदला (Elikā jhiripradalā)	Seed	4
14	*Coriandrum sativum L.*	Dhaniya	धानेयक: कुस्तुम्ब ुरु: (Dhāneyakaḥ kustumburuḥ)	Fruit	4
15	*Terminalia chebula Retz.*	Harad	वीरक: हरीतक: (Vīrakaḥ harītakaḥ)	Fruit Rind	4
16	*Swertia chirayita (Roxb.) H.Karst.*	Chirayata	घ ूर्णोभदलकम् ति क्तम् (Ghurṇobhadalakam tiktam)	Fruit	4
17	*Terminalia bellirica (Gaertn.) Roxb.*	Baheda	वीरक: अक्ष : (Vīrakaḥ akṣaḥ)	Fruit Rind	3
18	*Piper nigrum L.*	Marich	कणि का मरि चा (Kaṇikā maricā)	Fruit	3
19	*Piper longum L.*	Pippali	कणि काप ि प ्पली (Kaṇikā pippalī)	Fruit	3
20	*Phyllanthus emblica L.*	Amla	आमलक: धात्रीफल: (Āmalakaḥ dhātrīphalaḥ)	Fruit	3
21	*Piper retrofractum Vahl*	Chavya	कणि का गोलसपत्रा (Kaṇikā golasapatrā)	Stem	3
22	*Embelia ribes Burm.f.*	Vayavidang	वि डङ ्गक: कृमि घ ्न: (Viḍaṅ gakaḥ kṛmighnaḥ)	Fruit	3
23	*Scindapsus officinalis (Roxb.) Schott*	Gajpeepal	कण ाफलकम् पक्षवृन्तम् (Kaṇāphalakam pakṣavṛntam)	Fruit	3
22	*Zingiber officinale Roscoe*	Sonth	आर्द्रकम् सि तौष ्ठ म् (Ārdrakam sitauṣṭ ham)	Rhizomes	3
24	*Tribulus terrestris L.*	Gokharu	गोक्ष ुरक: त्रि श ूल: (Gokṣurakaḥ triśūlaḥ)	Stem	150
25	*Boerhavia diffusa L.*	Punarnava	प ुनर्नवक: रक्तकाण ्ड : (Punarnavakaḥ raktakāṇḍaḥ)	Plant	30
26	*Solanum lycopersicum* var. *lycopersicum*	Tomato extract (10% Lycopene)	य ुक्पञ ्चकम् रसप ूरम् (Yukpañcakam rasapūram)	Fruit	20
Fine powders of:					
27	*Mandoor Bhasma*	–	–	Mineral	30
28	*Lauh Bhasma*	–	–	Mineral	30
29	*Asphaltum punjabianum*	Shuddh Shilajit	–	Exudate	75
30	*Commiphora wightii (Arn.) Bhandari*	Shuddha Guggulu	गुग्गुलक: गण ्डिशाख : (Guggulakaḥ gaṇḍiśākhaḥ)	Resin	75

Excipients: Gum acacia (*Acacia arabica*), Talcum, Microcrystalline cellulose and Croscarmellose sodium have been used for formulating Prostogrit tablet

### 2.2 Ultra high performance liquid chromatography-photodiode array analysis of Prostogrit

Prominence-XR UHPLC system from Shimadzu, Japan, was utilized for the analysis. The system included a Quaternary pump (NexeraXR LC-20AD XR), a DAD detector (SPD-M20 A), an auto-sampler (Nexera XR SIL-20 AC XR), a degassing unit (DGU-20A 5R), and a column oven (CTO-10 AS VP). The sample was separated using a Shodex C18-4E (5µm, 4.6 × 250 mm) column through binary gradient elution. During the analysis, two solvents were used - Solvent A: 0.1% orthophosphoric acid adjusted to pH 2.5 with diethyl amine, and Solvent B: acetonitrile. The solvent system was set to follow a gradient elution process, starting with 5% B for 0–5 minutes, 5-25% B from 5–30 minutes, 25-45% B from 30–40 minutes, 45-70% B from 40–50 minutes, 70% B from 50–55 minutes, 70-85% B from 55–65 minutes, 85-5% B from 65–66 minutes, and finally 5% B from 66–70 minutes. The flow rate of the solvents was set at 1.0 mL/min. A 10 µL sample was injected for analysis, and the column temperature was kept at 35 °C. The wavelengths used were 240 nm for benzoic acid, guggulsterone E, and guggulsterone Z; 270 nm for gallic acid, 5-hydroxymethylfurfural (5-HMF), methyl gallate, and cinnamic acid; and 340 nm for piperine.

### 2.3 Experimental animals

The animal care and treatment procedures adhered to the guidelines provided by Committee for the Control and Supervision of Experiments on Animals (CCSEA), Department of Animal Husbandry and Dairying, Ministry of Fisheries, Animal Husbandry and Dairying, Government of India and the experimental protocol was approved by the Institutional Animal Ethics Committee of Patanjali Research Foundation, prior to the commencement of the experiment, vide protocol number PRIAS/LAF/IAEC-143/E1. Forty two male SD rats, aged between 4 to 5 weeks, were procured from Hylasco Bio-technology (India) Pvt. Ltd, a technology licensee of Charles River Laboratories and housed in a CCSEA registered vivarium (Registration No: 1964/PO/RC/S/17/CPCSEA) with a 12-hour light and dark cycle at a temperature of 22 ± 3 °C and relative humidity of 30-70%. They were provided with Purina Lab Diet (5L79; containing 18% protein), manufactured by PMI Nutrition International Ltd., USA, and sterile filtered water *ad libitum*.

### 2.4 Compound administration and disease induction

**Figure 1:**
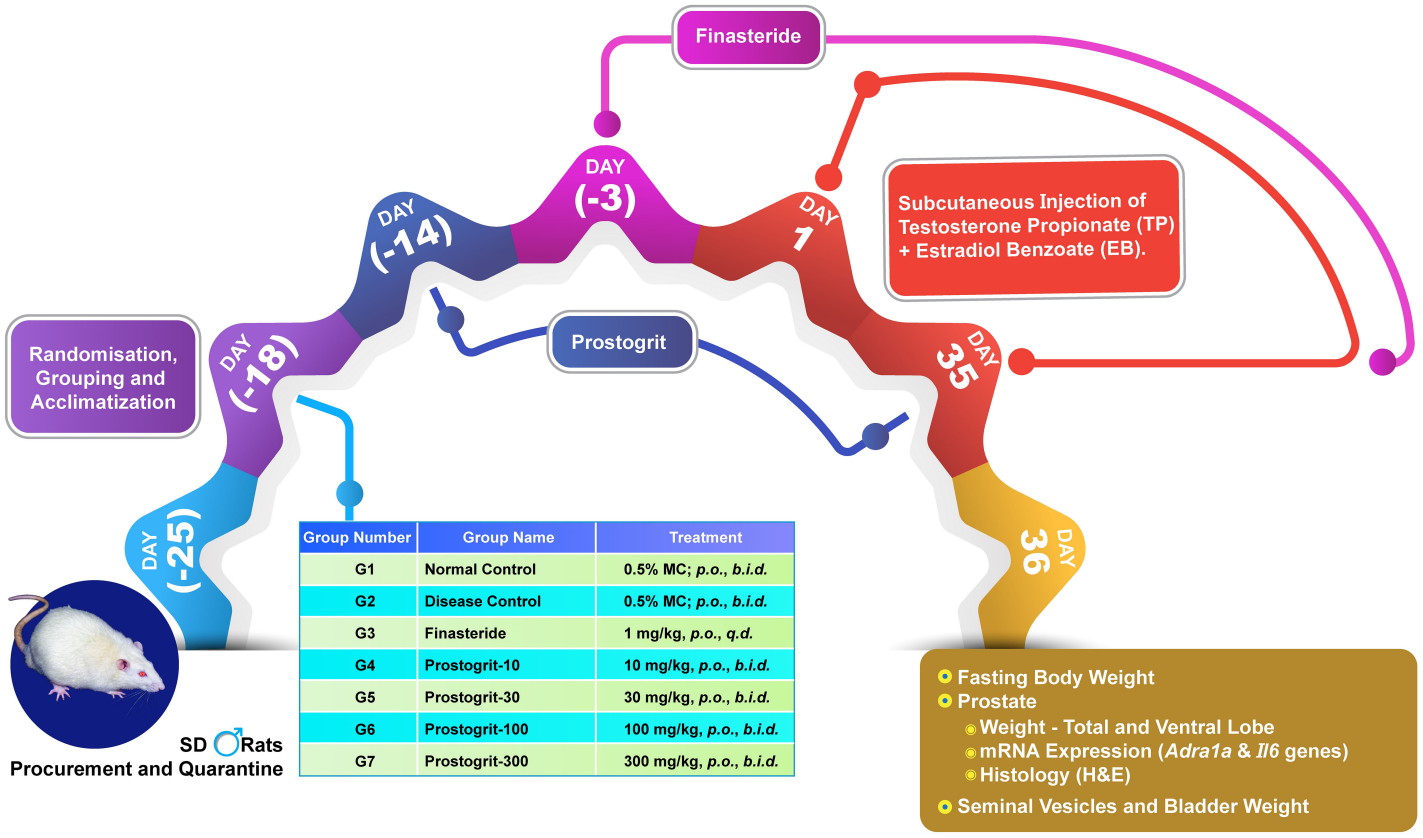
Schematic representation of assessment of Prostogrit in rat model of benign prostatic hyperplasia induced by a combination of testosterone propionate and Estradiol benzoate. Following the completion of the quarantine and acclimatization period, male Sprague-Dawley (SD) rats were orally administered Prostogrit prophylactically for 14-consecutive days. Throughout this experimental phase, the rats in the normal-control and disease-control groups received 0.5% methylcellulose orally, twice daily. In contrast, the rats receiving Prostogrit were given the formulation as a suspension in 0.5% MC by gavage twice daily, at the escalating doses of 10, 30, 100, and 300 mg/kg, twice daily. Upon completion of the prophylactic treatment, each animal was subcutaneously injected with 100 µL of a mixture containing testosterone propionate (TP; 2 mg/animal/day) and estradiol benzoate (EB; 0.02 mg/animal/day) for 35 consecutive days while the normal control group received sesame oil (vehicle for TP and EB). The finasteride-treated group received finasteride three days before disease induction at the dose of 1 mg/kg, as a gavage, once daily. After the final induction, the animals underwent overnight fasting before being anesthetized and euthanized. Subsequently, prostate tissue, seminal vesicles, and urinary bladder were collected, and their weights were recorded. Histological assessments were conducted on the half-ventral lobe of the prostate, with the second half-ventral lobe analyzed for mRNA expression of *Adra1a* and *Il6* genes.

Rats were allocated into seven groups, with six animals in each group (**Figure 1**). The first group was designated as normal control group, while second group was the disease control group. Both groups received 0.5% methylcellulose (MC), the vehicle for preparing suspensions of both Prostogrit and finasteride, *b.i.d.* by oral route. The third group served as the reference-control group and received finasteride (1 mg/kg, *q.d.*), orally. Groups 4 to 7, received Prostogrit as a gavage at the doses of 10, 30, 100 and 300 mg/kg, twice daily. The clinically equivalent dose of Prostogrit was calculated as 200 mg/kg/day (or 100 mg/kg, *b.i.d.*) in rats, derived from the human dose of 2000 mg/day using body surface area conversion (17), The remaining tested doses were from 1/10^th^ to 3 times of the therapeutically relevant dose and were included to obtain a probable dose-response. Animals received vehicle and Prostogrit orally for 14 days prophylactically and then concurrently with disease induction. Finasteride administration on the other hand, was initiated from three-days prior to disease induction and then continued along with the BPH induction regimen. Throughout the experiment, all compounds were administered orally in a dose volume of 5 mL per kg body weight. Body weight of animals was recorded once a week on the designated day, prior to gavage. Thereafter, required volume of vehicle or test drug suspension was calculated for each rat based on this most recent weekly weight. Subsequently, animals were administered the required volume of the treatment, intragastrically as per the most recent body weight.

After 14-days of prophylactic administration, the rats were subjected to induction of BPH using a slight modification of a previously published protocol (15). Animals allocated to the normal control group were injected 100 µL of sesame oil, subcutaneously for 35 consecutive-days. In contrast, the animals assigned to the other study groups were subcutaneously injected with a mixture of Testosterone propionate (2 mg/animal) and Estradiol benzoate (0.02 mg/animal) dissolved in sesame oil, at a ratio of 100:1 for 35-consecutive days.

### 2.5 Organ collection and weighing

The terminal body weights of all the overnight fasted rats were recorded on day 36. Subsequently, they were euthanized under an overdose of thiopentone (150 mg/kg, injected intraperitoneally) and their prostate, seminal vesicles and urinary bladders were excised. The ventral lobe of the prostate was carefully dissected out and then wet weights of all the harvested organs were immediately recorded. The relative organ weight was calculated by using the following formula:


$$Relative\;organ\;weight\;(\%)=\;\frac{Weight\;of\;the\;organ\;(g)\;\;}{Fasted\;body\;weight\;(g)}\;\times 100$$

### 2.6 Processing of the ventral prostate for histological analysis

Post-excision, one lobe of the ventral prostate was fixed in 10% neutral buffered formalin and subjected to standard in-house tissue processing procedures, following which, it was embedded in paraffin. From the obtained paraffin blocks, sections of thickness 3-5 µm were subsequently obtained. These sections were then transferred on to glass slides, deparaffinized and stained with hematoxylin and eosin (H&E) for histopathological analysis. Images were then captured at 100 times magnification. H&E-stained sections were consequently analyzed for epithelial proliferation, acinar hypertrophy and inflammatory cell influx by a veterinary histopathologist who was blinded to the various treatment groups. The severity of histopathological changes was scored using a semi-quantitative grading scale, which ranged from 0-4. The criteria for scoring were 0 = Nil; 1 = Minimal; 2 = Mild, 3 = Moderate and 4 = Severe (18). The individual lesion scores were finally added for all the animals and the summation was represented as total lesion score.

### 2.7 Quantitative real time PCR for mRNA expression of *Adra1a* and *Il6* genes in prostate tissue

The second lobe of the ventral prostate from five animals per group was immersed in RNAProtect reagent and the samples were processed for mRNA expression of *Adra1a* and *Il6* genes by qRT-PCR. The total RNA was extracted from rat prostate tissues using TRIzol reagent. 1μg total RNA was utilized for cDNA synthesis using Verso cDNA synthesis kit. Quantitative real-time PCR (qRT-PCR) was performed using qTOWER3 G instrument (Analytik-Jena, Jena, Germany). A 10 μL qRT-PCR reaction mixture containing 10–50 ng of cDNA, 0.5 μL (10 pM) each of forward and reverse primers, and 5 μL of PowerUp SYBR Green Master Mix was used. qRT-PCR cycle conditions were as follows: 95°C for 5 min, followed by 40 cycles of denaturation at 95°C for 30 s, annealing at 58°C for 30 s, extension at 72°C for 30 s and final extension at 72°C for 5 min. β-actin gene (*Actb*) was used as a reference for normalizing the data. The fold change of each studied gene was calculated using 2^−ΔΔCt^ method (19). The sequence of forward (FW) and reverse (RV) primers are as follows:

*Actb* FW: 5′-GGGAAATCGTGCGTGACATT-3’
*Actb* RV: 5′-GCGGCAGTGGCCATCTC-3’
*Adra1a* FW: 5′-AATGCTTCGGAAGGCTCCAA-3’
*Adra1a* RV: 5′-CCCCAAGCAGAATGGCCTTA-3’
*Il6* FW: 5′-TCCTACCCCAACTTCCAATGCT-3’
*Il6* RV: 5’-TTGGATGGTCTTGGTCCTTAGCC-3’

The complete study design covering all the above mentioned experiments has been depicted in **Figure 1**.

### 2.8 Statistical analysis

The data for the parameters of the study were compiled from all the study groups and expressed as mean ± standard error of mean (SEM) and thereafter, statistical analysis was conducted using GraphPad Prism version 10.1.1 software (GraphPad Software, USA). One-way analysis of variance (ANOVA) followed by Dunnett’s multiple comparison *post-hoc* test was used to compute the statistical differences between the means. Effect sizes and 95% confidence intervals were calculated where applicable. A p value < 0.05 was considered to be statistically significant.

## 3 Results

### 3.1 Prostogrit is enriched with phytometabolites with known pharmacological effects against BPH

**Figure 2:**
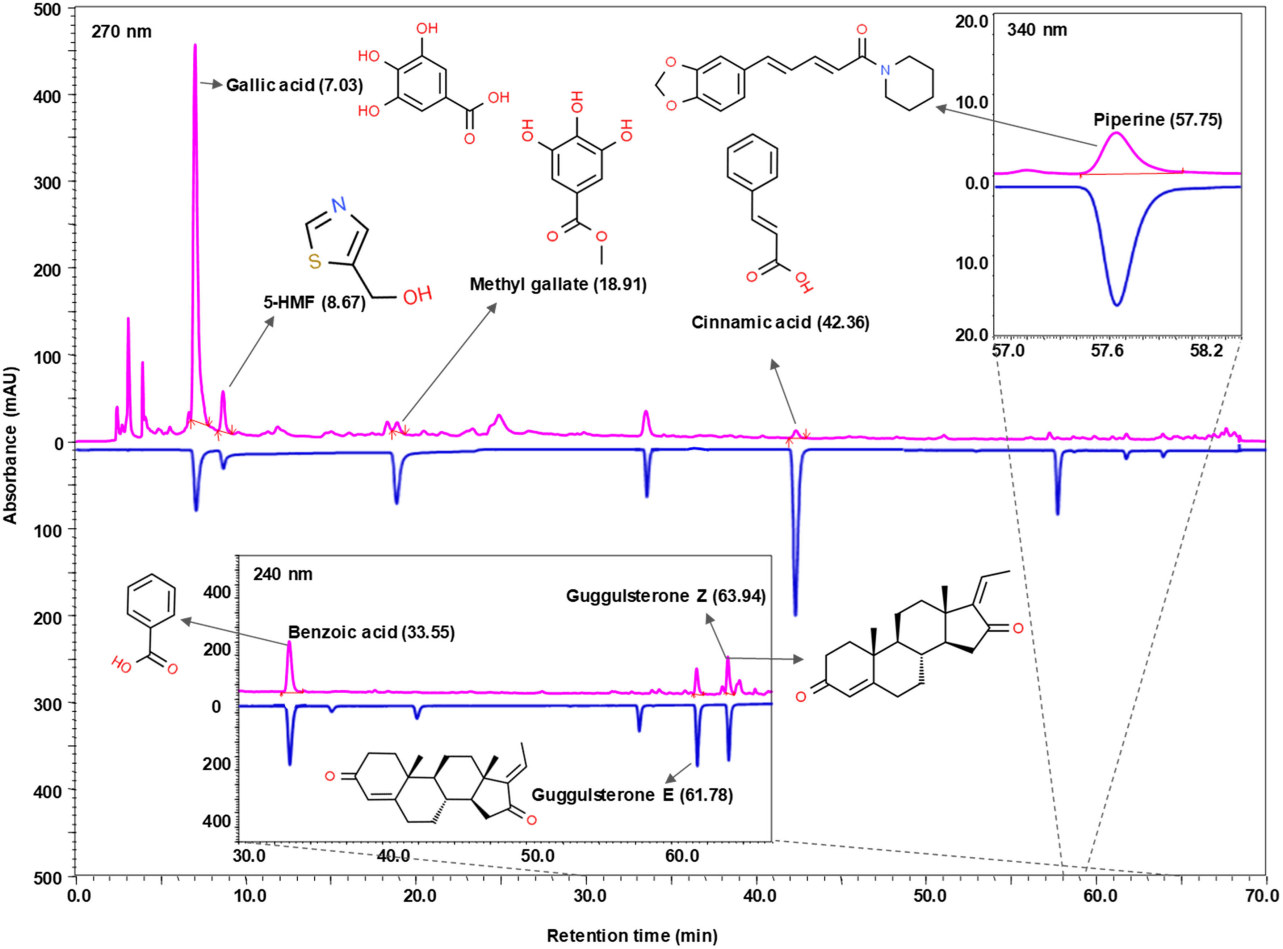
Phytochemical analysis of Prostogrit. Prostogrit underwent phytochemical analysis on UHPLC-PDA platform (chromatogram portrayed in pink) and compared with pure reference standards (chromatogram depicted in blue). Eight distinct compounds were identified and quantified in Prostogrit, as highlighted in **Table 2**. Benzoic acid, guggulsterone E, and guggulsterone Z were identified at 240 nm, whereas gallic acid, 5-hydroxymethylfurfural (5-HMF), methyl gallate, and cinnamic acid, were detected at 270 nm. Piperine was identified at 340 nm. The chemical structures of the identified phytochemicals have been incorporated alongside the chromatograms, with sources cited from www.chemspider.com.

Phytochemical analysis of Prostogrit by utilization of the UHPLC-PDA platform, revealed presence of eight phytometabolites in the tested sample (**Figure 2**). Through this analytical method, a direct comparison of the chromatogram of Prostogrit with that of the pure standard compounds, facilitated the detection of the respective phytocompounds. Subsequently, all the identified phytocompounds were subjected to quantification and it was ascertained that each milligram of the herbo-mineral medicine contained benzoic acid (0.159 µg), guggulsterone E (0.417 µg), guggulsterone Z (0.655 µg), gallic acid (4.073 µg), 5-hydroxymethylfurfural (1.177 µg), methyl gallate (0.122 µg), cinnamic acid (0.043 µg), and piperine (0.018 µg) (**Table 2**).

**Table 2:** Phytometabolites identified and quantified in Prostogrit by UHPLC-PDA analysis, as depicted in **Figure 2**.

S. no.	Phytometabolite detected	Molecular weight (g/mol)	Content in prostogrit (µg/mg)	Retention time (minutes)
1	Gallic acid	170.12	4.073	7.03
2	5-hydroxymethyl furfural (5-HMF)	126.11	1.177	8.67
3	Guggulsterone Z	312.45	0.655	63.94
4	Guggulsterone E	312.45	0.417	61.78
5	Benzoic acid	122.12	0.159	33.55
6	Methyl gallate	184.15	0.122	18.91
7	Cinnamic acid	148.16	0.043	42.36
8	Piperine	285.34	0.018	57.75

### 3.2 TP+EB administration impairs the body weight gain in rats

**Figure 3:**
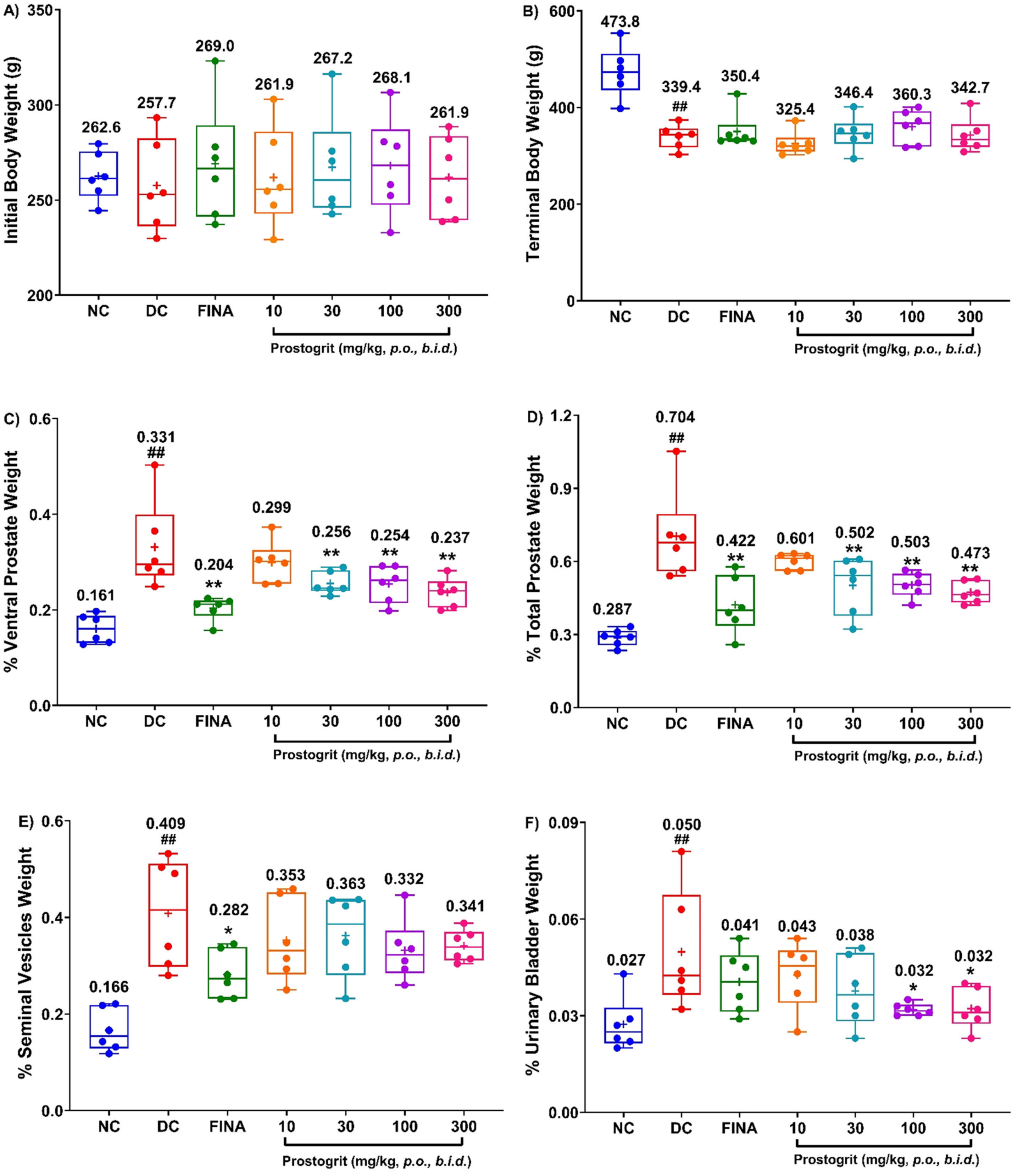
Effects of Prostogrit on body weight and relative weights of ventral prostate, total prostate, seminal vesicles and urinary bladder. Body weights of the rats were recorded at initiation as well as on the day of termination of the experiment and the prostate, seminal vesicles and urinary bladder were excised as elaborated in the Materials and Methods section. **(A)** Represents the body weight of the animals at study initiation. **(B)** Represents fasting body weights at termination. **(C)** Depicts relative organ weight of the ventral prostate. **(D)** Represents relative organ weight of the total prostate. **(E)** Represents the relative organ weight of the seminal vesicles. **(F)** Represents the relative organ weight of the urinary bladder. Data is presented as box and whisker plots (n=6 animals per group). The mean value for the plots has been denoted by the + symbol and is numerically represented above the box for each experimental group. Statistical analysis was performed by employing one-way analysis of variance, followed by Dunnett’s multi-comparison *post-hoc* test. ## represents significant (p < 0.01) difference between disease control and normal control group, and *, ** represents significant difference with p < 0.05, and p < 0.01, respectively, between disease control and treatment groups. NC, normal control; DC, disease control; FINA, finasteride-1 mg/kg, once daily.

The box and whisker plots of the mean (± SEM) body weights of the rats at the initiation of the study are represented in **Figure 3A**, whereas those at study termination are depicted in **Figure 3B**. At the initiation of the test article administration, the body weights across all the study groups were not statistically different (**Figure 3A**). However, on the day of study termination, the body weights of the animals allocated to the disease-control group were discerned to be significantly lower as compared to the normal control group (p < 0.01; **Figure 3B**). Both Prostogrit and finasteride did not prevent the decrease in body weights induced by TP+EB.

### 3.3 Prostogrit modulates TP+EB-induced increase in relative weights of prostate, seminal vesicles and urinary bladder

The box and whisker plots of the relative weights of the ventral lobes of prostate gland of all the experimental groups are depicted in **Figure 3C** and those of the total prostate are portrayed in **Figure 3D**. In the normal control group, the mean (± SEM) relative weights of the ventral prostate were 0.161 ± 0.012%, whereas those of the total prostate were 0.287 ± 0.014%. Repeated subcutaneous administration of a mixture of TP+EB for five-consecutive weeks, led to a significant increase in the mean relative organ weights of ventral (0.331 ± 0.038%) as well as that of the mean total prostate (0.704 ± 0.075%), when compared to the normal control group (p < 0.01, **Figures 3C, D**). This observed outcome demonstrates successful generation of the rat model of BPH. Method control drug, finasteride decreased the mean relative organ weights of ventral (0.204 ± 0.009%) as well as that of the mean total prostate (0.422 ± 0.048%). The observed results were statistically significant when compared to the disease-control group (p < 0.01; **Figures 3C, D**, respectively), signifying validation of the experimental model. In this experimental model, Prostogrit when administered by oral route, ameliorated TP+EB-induced increase in the relative organ weight of the ventral lobe of the prostate as well as that of the whole prostate in a dose-dependent fashion. The calculated mean relative ventral prostate weights in animals treated with Prostogrit at the doses of 10, 30, 100 and 300 mg/kg, *b.i.d.* were 0.299 ± 0.018%, 0.256 ± 0.001%, 0.254 ± 0.016% and 0.237 ± 0.012%, respectively. The observed effects of Prostogrit were significantly different when compared to the disease control group at the doses of 30, 100 and 300 mg/kg, *b.i.d.* (p < 0.01 at **Figure 3C**). Further, the mean relative total prostate weights in rats administered with Prostogrit at the doses of 10, 30, 100 and 300 mg/kg, *b.i.d.* were 0.601 ± 0.013%, 0.502 ± 0.048%, 0.503 ± 0.021 and 0.473 ± 0.018%, respectively. Similar to the obtained results for ventral prostate, Prostogrit also demonstrated statistically significant reductions in the relative total prostate weights at the doses of 30, 100 and 300 mg/kg, *b.i.d.* (p < 0.01 *vs*. disease control group, **Figure 3D**).

The relative weights of the seminal vesicles of rats, represented as box and whisker plots, from all the study groups are illustrated in **Figure 3E**. In the normal control group, the mean (± SEM) relative weights of the seminal vesicles were 0.166 ± 0.018%. Similar to the observed increase in prostate weights, TP+EB also induced a significant increase in mean relative weights of the seminal vesicles (0.409 ± 0.046%), when compared to normal control group (p < 0.01, **Figure 3E**). The average relative weight of the seminal vesicles was reduced by finasteride treatment (0.282 ± 0.020%) and the effect was significantly different when compared to the normal control group < 0.05, **Figure 3E**). Prostogrit, demonstrated a tendency to reduce the relative seminal vesicles weight as the calculated average relative weight of seminal vesicles in rats treated with Prostogrit at the doses of 10, 30, 100 and 300 mg/kg were 0.353 ± 0.035%, 0.363 ± 0.035%, 0.332 ± 0.026% and 0.341 ± 0.014% respectively. However, the observed effects of Prostogrit were not statistically different from the disease control group.

Furthermore, the plots of the relative weights of the urinary bladders of animals allocated to the various treatment groups are shown in **Figure 3F**. The mean (± SEM) relative urinary bladder weights of the animals allocated to the normal control group was 0.027 ± 0.003%. As noted for prostate gland and seminal vesicles, continual TP+EB administration for 35-consecutive days also demonstrated a significant increase in the mean relative weight of the urinary bladder (0.050 ± 0.008%), as compared to normal control group (p < 0.01, **Figure 3F**), which tended to be lowered by finasteride (0.041 ± 0.004%) at the tested dose of 1 mg/kg, *p.o.* However, the observed effect with finasteride treatment was not significantly different from the disease control group. Prostogrit on the other hand demonstrated a dose- dependent reduction in TP+EB induced augmentation of relative urinary bladder weight. The calculated relative weights of the urinary bladders of animals treated with Prostogrit at the doses 10, 30, 100 and 300 mg/kg, *b.i.d.* were 0.043 ± 0.004%, 0.038 ± 0.004%, 0.032 ± 0.001% and 0.032 ± 0.003%, respectively. Statistically significant diminution as compared to the disease control group were evident at the doses of 100 and 300 mg/kg, *b.i.d.* (p < 0.05, **Figure 3F**).

**Figure 4:**
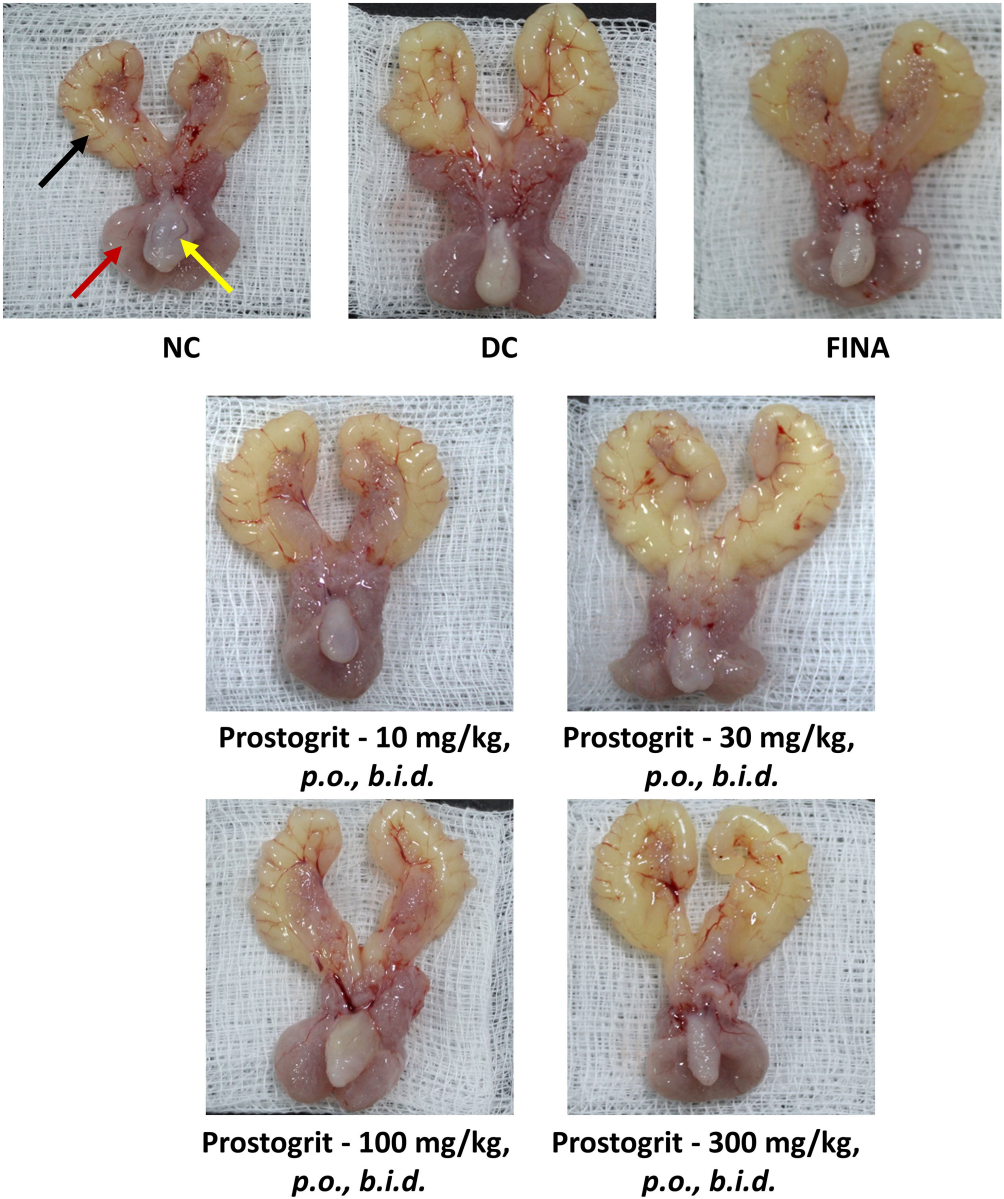
Prostogrit decreases TP+EB-induced increase in prostate mass. Representative images of the lower urinary tract of animals from each of the study groups. Red arrow depicts the ventral prostatic lobe, black arrow indicates the seminal vesicle and the yellow arrow represents the urinary bladder.NC, normal control; DC, disease control; FINA, finasteride (1 mg/kg, q.d.).

In addition to the relative organ weight data, the representative macroscopic images of the prostate gland, seminal vesicles and urinary bladder excised from the animals allocated to the various experimental groups have been depicted in **Figure 4**.

### 3.4 Prostogrit alleviates TP+EB-induced histological abnormalities in the prostate

**Figure 5:**
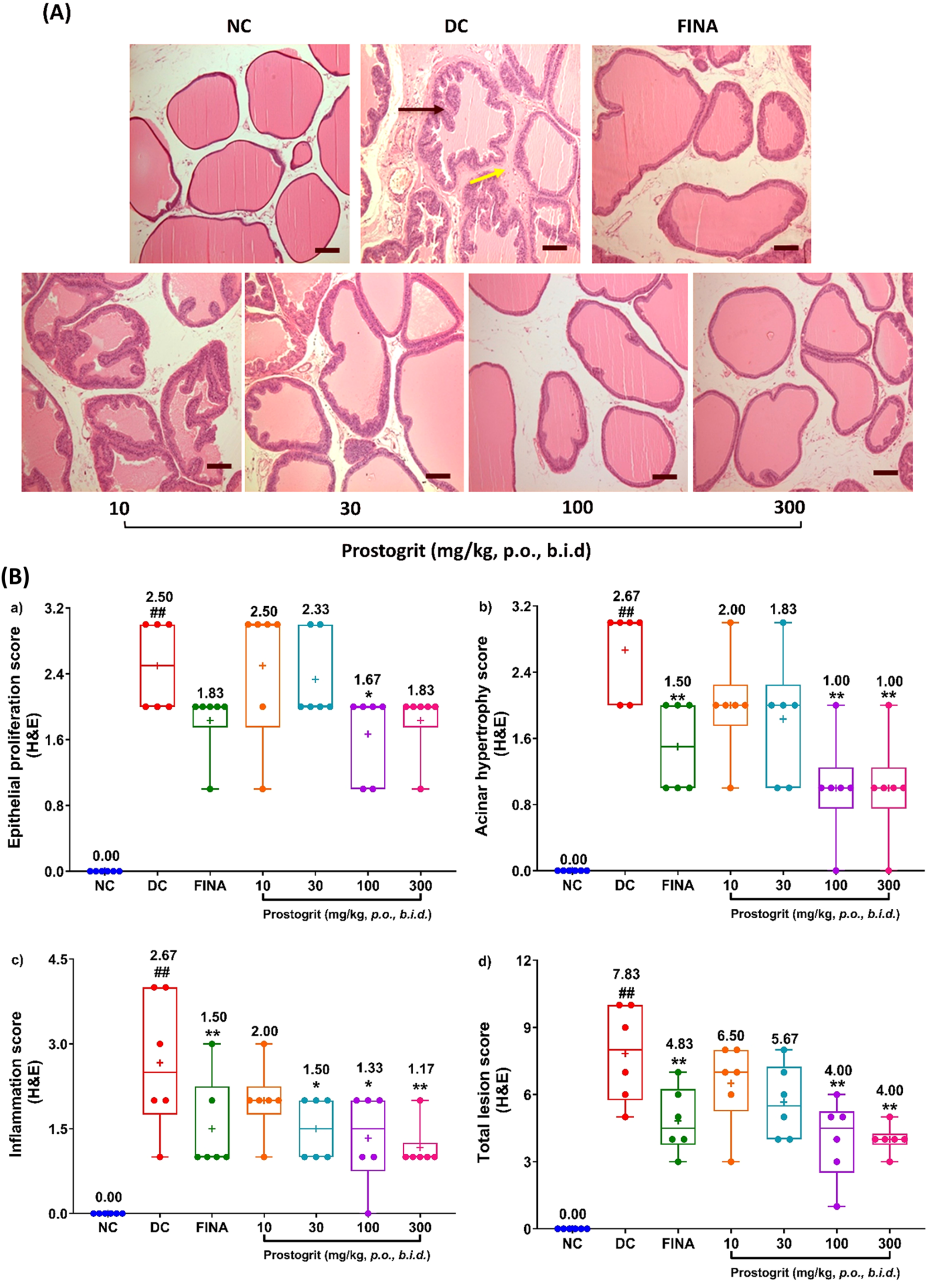
Prostogrit reduces TP+EB induced histological alterations. Ventral prostatic lobes were excised from the harvested prostate and processed for hematoxylin and eosin (H&E) staining. **(A)** depicts the representative photomicrographs of the prostate (× 100) from each of the study groups. Scale bar = 50 µm. The black arrow indicates hyperplastic changes in the epithelium of the acinar cells, whereas the yellow arrow represents the stromal inflammation. **(B)** Depicts the semi-quantitative scores for a) epithelial hyperplasia b) acinar hypertrophy c) stromal inflammation and d) total lesion score. Data is presented as box and whisker plots (n=6 animals per group). The mean value for the plots has been denoted by the + symbol and is numerically represented above the box for each experimental group. Statistical analysis was performed by employing one-way analysis of variance, followed by Dunnett’s multi-comparison *post-hoc* test. ## represents significant (p < 0.01) difference between disease control and normal control group, and *, ** represents significant difference with p < 0.05, and p < 0.01, respectively, between disease control and treatment groups. NC, normal control; DC, disease control; FINA, finasteride (1 mg/kg, q.d.).

Microscopic examinations of the ventral prostates from all the experimental groups have been presented in **Figure 5A**, whereas the box and whisker plots of the semi-quantitative histopathological lesion scores have been depicted in **Figure 5B**. Rats allocated to the normal control group exhibited columnar epithelium with regular acini. The acinar lumen contained only a small number of papillary folds and was filled with prostatic secretions. The matrix was observed to be normal, representing the typical normal histology of the prostate. Contrastingly, prostate tissue from disease control group displayed epithelial proliferation due to distinct epithelial hyperplasia, with numerous intraluminal folds, lumen narrowing, thereby leading to reduced lumen volume along with the infiltration of inflammatory cells (**Figure 5A**). To recapitulate, repeated administration of TP+EB lead to a significant alteration in the microscopic structure of the prostate manifesting as epithelial proliferation, acinar hypertrophy and influx of inflammatory cells, when compared to normal control group (**Figure 5A**). The mean (± SEM) scores for the microscopic lesions in rats from the normal control group were 0.00 ± 0.00. Conversely, the mean semi-quantitative histopathological lesion scores in disease control rats were 2.50 ± 0.22 for epithelial proliferation; 2.67 ± 0.21 for acinar hypertrophy and 2.67 ± 0.49 for inflammatory cell influx. The mean combined or total lesion score in disease control group was 7.83 ± 0.87. All the obtained lesion scores were significantly different when compared to the normal control group (p < 0.01, **Figure 5**, Panel B (a-d). The average lesion scores in animals treated with method control drug, finasteride were 1.83 ± 0.17 for epithelial proliferation; 1.50 ± 0.22 for acinar hypertrophy; 1.50 ± 0.34 for infiltration of inflammatory cell and the total lesion score was 4.83 ± 0.60. The epithelial proliferation score in finasteride treated animals was lower but not statistically significant when compared to the disease control group (**Figure 5B**). Nevertheless, the acinar hypertrophy, inflammation and total lesion scores in finasteride-treated animals were significantly different as compared to the disease control group.

Prostogrit, administered orally attenuated TP+EB-evoked epithelial proliferation. The mean scores for epithelial proliferation in ventral prostate of rats treated with Prostogrit at the doses of 10, 30, 100 and 300 mg/kg, *b.i.d.* were 2.50 ± 0.34, 2.33 ± 0.21, 1.67 ± 0.21 and 1.83 ± 0.17, respectively. The detected effects were statistically significant when compared to the disease control group at the therapeutically relevant dose of 100 mg/kg, *b.i.d.* (p < 0.05, **Figure 5B**). Similar to epithelial proliferation, Prostogrit also demonstrated amelioration of acinar hypertrophy. The mean semi-quantitative lesion scores for this observed parameter in rats treated with Prostogrit, at the doses of 10, 30, 100 and 300 mg/kg, *b.i.d.* were 2.00 ± 0.26, 1.83 ± 0.31, 1.00 ± 0.26 and 1.00 ± 0.26, respectively. The obtained effects were dose-dependent and significant outcomes were evident at 100 and 300 mg/kg, *b.i.d.* (p < 0.01, **Figure 5B**), as compared to the disease. Likewise, Prostogrit also demonstrated inhibition of the influx of inflammatory cells induced by TP+EB (**Figure 5A**). The average inflammatory cell influx scores in animals administered with Prostogrit at the doses of 10, 30, 100 and 300 mg/kg, *b.i.d.* were 2.00 ± 0.26, 1.50 ± 0.22, 1.33 ± 0.33 and 1.17 ± 0.17, respectively. The effects of Prostogrit on disease-associated inflammatory cell infiltration were dose-dependent and statistically significant when compared to the disease control group at the doses of 30, 100 and 300 mg/kg, *b.i.d.* (p < 0.05 for 30 and 100 mg/kg *b.i.d.* and p < 0.01 at 300 mg/kg, *b.i.d.*, **Figure 5B**). Finally, the summation of all the evaluated lesion scores revealed that Prostogrit mitigated TP+EB evoked pathological alterations in the prostate. The mean combined lesion scores in rats treated with Prostogrit at the doses of 10, 30, 100 and 300 mg/kg, b.i.d. were 6.50 ± 0.76, 5.67 ± 0.67, 4.00 ± 0.73 and 4.00 ± 0.26. Statistically significant effects for total lesion score were apparent at the therapeutically relevant dose of 100 mg/kg, *b.i.d.* as well as 300 mg/kg, *b.i.d.* (p < 0.01, **Figure 5B**).

### 3.5 In rat prostate tissue, Prostogrit moderates TP+EB-induced mRNA expression of *Adra1a* and *Il6* genes

**Figure 6:**
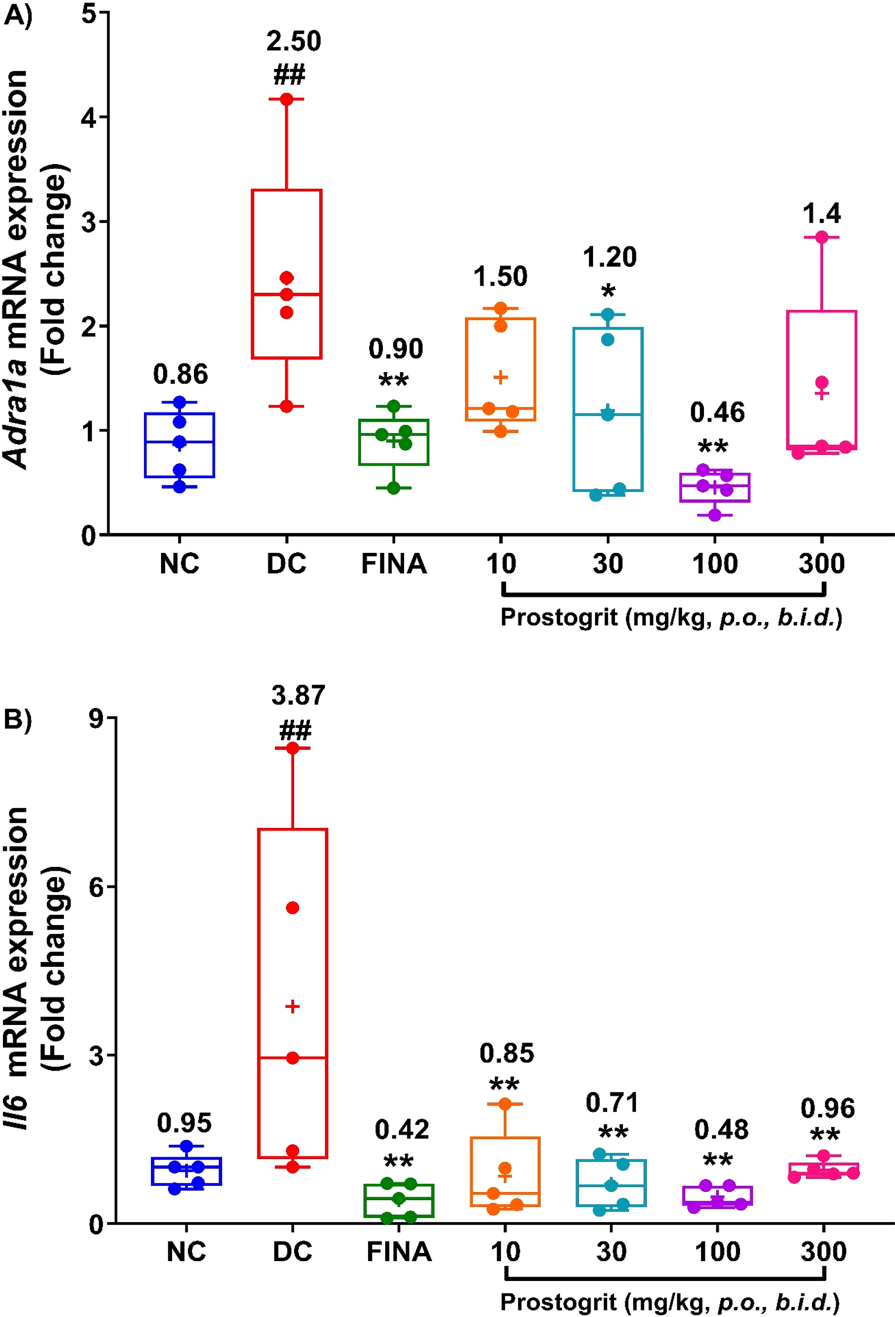
Prostogrit reduces mRNA expression of *Adra1a* and *Il6* gene in ventral prostate. The mRNA expression of the studied markers was assessed by qRT-PCR. **(A)***Adra1a* gene. **(B)***Il6 gene*. Data is presented as box and whisker plots (n=5 animals per group). The mean value for the plots has been denoted by the + symbol and is numerically represented above the box for each of the experimental groups. Statistical analysis was performed by employing one-way analysis of variance, followed by Dunnett’s multi-comparison *post-hoc* test. ## represents significant (p < 0.01) difference between disease control and normal control group, and *, ** represents significant difference with p < 0.05, and p < 0.01, respectively, between disease control and treatment groups. NC, normal control; DC, disease control; FINA, finasteride (1 mg/kg, q.d.).

In the present study, we also assessed the impact of Prostogrit on the mRNA expression of *Adra1a* as well as that of *Il6* genes in the prostate. The CT (mean ± SEM) values of *Actb* gene (housekeeping gene) for different groups were: 17.77 ± 0.29 for group 1; 17.44 ± 0.18 for group 2; 17.81 ± 0.19 for group 3; 17.64 ± 0.25 for group 4; 17.45 ± 0.21 for group 5; 17.98 ± 0.27 for group 6 and 18.55 ± 0.16 for group 7, signifying that *Actb* is suitable for using as a housekeeping gene, in this study. The box and whisker plots of the fold changes of mRNA expression of *Adra1a* gene is illustrated in **Figure 6A** and those for *Il6* gene is shown in **Figure 6B**. In normal control group the mean (± SEM) fold change for *Adra1a* mRNA expression was 0.86 ± 0.15. TP+EB administration augmented the mRNA expression of *Adra1a* gene as the observed fold change in the disease control group was 2.50 ± 0.48. This observed increase was significantly different when compared to the normal control group (p < 0.01, **Figure 6A**). Finasteride treated animals demonstrated an average fold change of 0.90 ± 0.13 and its effect was significantly different from the disease control group (p < 0.01, **Figure 6A**). The observed effect on mRNA expression of *Adra1a* gene was inhibited by Prostogrit. The mean fold changes in animals treated with Prostogrit at the doses of 10, 30, 100 and 300 mg/kg, *b.i.d.* were 1.50 ± 0.24, 1.20 ± 0.36, 0.46 ± 0.08 and 1.4 ± 0.39, respectively. Statistically significant effects were noted at the doses of 30 and 100 mg/kg, *b.i.d.* (p < 0.05 at 30 mg/kg, *b.i.d.* and p < 0.01 at 100 mg/kg, *b.i.d.*, **Figure 6A**). Further, in the animals assigned to the normal control group the mean fold changes in the mRNA expression of *Il6* gene was 0.95 ± 0.13. Contrastingly, in rats allocated to the disease control group, the average fold change was augmented to 3.87 ± 1.41, which was significantly different from the normal control group (p < 0.01, **Figure 6B**). In rats that were administered finasteride the mean mRNA expression of the *Il6* gene was significantly lowered to 0.42 ± 0.14 (p < 0.01 *vs.* disease control group, **Figure 6B**). Similarly, Prostogrit also attenuated TP+EB-induced increased mRNA expression of *Il6* gene. The observed mean fold changes in the rats treated with Prostogrit at the doses of 10, 30, 100 and 300 mg/kg, *b.i.d.* were 0.85 ± 0.34, 0.71 ± 0.19, 0.48 ± 0.09 and 0.96 ± 0.07. Significant reduction of the *Il6* mRNA expression by Prostogrit were noted at all the tested doses, when compared to the disease control group (p < 0.01, **Figure 6B**).

## 4 Discussion

In the current study, we successfully generated an animal model of BPH by exogenous administration of TP+EB, reflected by a significant increase in the relative weights of prostate, seminal vesicles and urinary bladder of rats. In addition to the evident macroscopic changes, the microscopic examination of prostate gland revealed structural changes, namely epithelial proliferation, hypertrophy of the acini and stromal inflammation. Additionally, we noticed an increased mRNA expression of *Adra1a* and *Il6* genes, which are relevant to pathogenesis of BPH and the associated BPS. For the purpose of model validation, we employed finasteride as a method or reference control drug. Finasteride, a 5ARI is widely used clinically for the treatment of BPS, owing to its proven disease modifying effects. In our study, finasteride could significantly moderate the gross manifestations of BPH-like disease phenotype in the prostate gland and the seminal vesicles of experimental animals. Finasteride was also effective in mitigating the microscopic alterations associated with BPH induction. In addition, it could also decrease the mRNA expression of the studied genes. We did not perform a dose response study with finasteride as our aim was to solely validate the animal model, thereby rendering it suitable for the *in vivo* pharmacological evaluation of the test article.

Further, in this validated model of BPH, we evaluated the preclinical effects of Prostogrit, which is a novel, Ayurvedic herbo-mineral medicine, indicated for the management of BPS. The current study was conceived as a proof of concept of the pharmacological effects of Prostogrit. Accordingly, Prostogrit was administered to the rats in a prophylactic manner, fourteen days prior to and then concurrent with disease induction with TP+EB. The results of this study revealed that, Prostogrit could significantly decrease the relative weights of the ventral prostate, total prostate and urinary bladder in a dose-dependent manner. It demonstrated a tendency to reduce the relative seminal vesicle weights as well. Additionally, Prostogrit could also ameliorate the TP+EB induced histopathological changes in the ventral lobe of the prostate like epithelial proliferation, acinar hypertrophy and infiltration of inflammatory cells in the prostatic stroma. Furthermore, Prostogrit could also reduce the TP+EB induced increased mRNA expression of *Adra1a* and *Il6* genes. These obtained outcomes indicate that Prostogrit possesses pharmacodynamic effects in an animal model of BPH.

BPS management remains challenging even after advancements in its pharmacotherapeutic and surgical management. Of these challenges, management of lower urinary tract symptoms (LUTS) are particularly bothersome in patients. LUTS arise as a consequence of the physical compression of the urethra by the enlarged prostatic mass. This leads to voiding symptoms, manifesting as a perception of incomplete urinary bladder emptying, straining to void urine, hesitation in urination as well as a weak urinary stream. Another component of LUTS includes storage symptoms characterized by increased episodes of nocturnal urination, frequent urination, dysuria and the urgency to urinate (20). The exact etiology remains unclear, but the disproportion of sex hormones plays a significant role (21), with chronic inflammation (22) and oxidative stress (23) being the other pathophysiological contributors. Contemporary prescription medicines, which form the cornerstone of the pharmacologic management of BPS include 5α-reductase inhibitors (5ARIs) and α_1_-adrenoceptor antagonists (11, 12). The prostate-specific Type II 5α-reductase enzyme catalyzes the conversion of testosterone to dihydrotestosterone (DHT) within the prostate, which is an essential regulator of prostate epithelial cell proliferation and function. 5ARIs like finasteride hinder conversion of testosterone to DHT. Consequently, reduction of the intra-prostatic levels of DHT by finasteride could lead to lowered occupation of nuclear receptors thereby inhibiting its effect on the growth and cellular function of the prostate (24). Clinical improvements with 5ARI therapy include a reduction in the prostatic volume and relief from the obstructive and irritative symptoms of BPS. Nonetheless, clinical improvements are observed after six months of initiation of the 5ARI treatment (25). α_1_-adrenoceptor antagonists on the other hand directly block the α_1_-adrenoceptor in the prostate and the urethra of patients, thereby relaxing the smooth muscle of the prostate and urinary bladder neck, broadening the urethral lumen, and facilitating the urine flow (26). From a safety perspective, the 5ARIs are associated with adverse effects such as decreased libido, ejaculatory disorder, gynecomastia and impotence (27), while α_1_-adrenoceptor antagonists are also associated with orthostatic hypotension, dizziness and abnormal including retrograde ejaculation (28). The limitations of the current standards of care of BPS, spurs the need for the development of newer therapies to address the enlarged prostatic size, the ensuing urethral obstruction as well as LUTS. Additionally, safer agents ideally demonstrating minimal to no treatment limiting adverse effects are desirable. In this light, a possible approach is discovery and development of novel plant-based medicines. Prostogrit, contains phyto- and mineral constituents with anti-inflammatory, antioxidant and tissue-repairing capabilities. Moreover, the constituents of Prostogrit have been used for millennia in traditional systems of medicine and are consequently anticipated to be safe for therapeutic use. Based on this foundation, Prostogrit has been specifically developed for the management of BPS, the clinical manifestation of the underlying BPH.

The rodent model of BPH induced by repeated injections of TP+EB has been used to decipher the pathophysiological mechanisms associated with the development of BPH as well as for the investigation of pharmacological interventions, preclinically (15, 16). Hence we selected this model to investigate the pharmacological effects of Prostogrit.

A noteworthy finding of the study was an impaired body weight gain of animals injected with TP+EB, as evidenced by lower terminal body weights of rats, when compared to sesame-oil alone administered rats. This outcome is in agreement with a previously conducted study and may be explained by exogenous testosterone-induced increased daily activity in rats as well as an augmented ratio of lean body mass/fat body mass (15).

The data from previously reported studies have established that increased prostate volume and weight is used as one of the significant markers to indicate the development of BPH (29). Furthermore, the prostate index, which is also derived from the prostate weight is a critical marker of BPH development (30). Since Prostogrit reduced the relative organ weight of the target organ, it indicates that Prostogrit has the potential to reduce the cardinal gross manifestation of BPH, in clinical conditions.

Exogenous androgen stimulation is also expected to increase the weight of the seminal vesicles and the same has been previously reported as well (31). In the current study also, an increase in relative seminal vesicle weight was observed, which tended to be lower in the Prostogrit treated groups.

The obstruction of urinary bladder outlet is one of the central clinical manifestation of BPS and long-term obstruction can lead to its hypertrophy as well as detrusor hyperplasia, finally culminating in the increase in urinary bladder weight. This phenomenon has been reported in clinical scenarios, *in vitro* and *in vivo* studies. The increase in urinary bladder weight could be regarded as an indicator of the progression of BPS, with a high correlation to the LUTS associated with the disease, especially detrusor over activity (32). Since, Prostogrit could efficiently ameliorate the increase in urinary bladder weight in the current study, this experimental outcome clearly points towards the potential of Prostogrit to address the management of LUTS in clinical conditions. Although this study was not designed to directly compare the *in vivo* efficacies of Prostogrit and finasteride, the observed effects allude to the possibility that Prostogrit may demonstrate pharmacologically activity in remedying the increase in urinary bladder weight, preclinically. However, well-designed comparative studies are needed to substantiate this claim.

The macroscopic enlargement of the prostate in BPS is an outcome of the underlying ultrastructural modifications within the prostate. In the present study, in the disease control group, we noticed epithelial hyperplastic changes in the acinar cells of the prostate. In addition, presence of numerous acinar epithelial folds was also detected, protruding towards the lumen of the acini, which consequently lead to an increase the acinar surface area. Accordingly, this observed histopathological anomaly has been designated as acinar hypertrophy. Furthermore, infiltration of inflammatory cells was also observed in the prostatic stroma. The above findings are in agreement with the studies that have used TP+EB to generate the rodent model of BPH (15, 16). In the current study, Prostogrit was able to reduce the TP+EB induced histological changes, suggesting that is has the preclinical potential to ameliorate the underlying pathological basis of the disease.

Sympathetic nerves innervate the smooth muscles of the prostate, anatomically located in the prostatic stroma. Noradrenaline released by these nerves act on the functionally relevant α_1A_-adrenoceptors, present on the surface of the prostatic smooth muscle cells, thereby regulating the prostatic tone. However, in BPS, prostatic smooth muscle proliferation is observed as a consequence of the long-term exposure to androgens. Consequently, these proliferative changes increase the prostatic size and statically obstructs the prostatic portion of the urethra. However, upregulation of α1A-adrenoceptors, also observed in the disease may increase the prostatic tone, thereby further contributing to the observed urethral constriction. This founds the rationale for the symptomatic use of α_1_ adrenoceptor antagonists in BPS, which act in part by blocking these receptors and relieving the increased prostatic tone (33, 34). Experimental assessments in animal models of BPH induced by testosterone and estradiol have demonstrated the upregulation of the α_1A_-adrenoceptors in the prostate tissue both at the protein (30) as well as the mRNA level (15, 31). It is noteworthy to state that in the current study, in accordance with the published literature evidence, we observed an enhanced mRNA expression of *Adra1a* gene in the prostate as well. Since, Prostogrit could attenuate the mRNA expression of *Adra1a*, it points towards the potential utility of the herbo-mineral medicine in relieving the dynamic obstruction of the prostate.

It has been well established that the prostatic infiltrates of BPS patients comprise of inflammatory cells, including neutrophils lymphocytes and macrophages (35). Further, several clinical studies support the correlation between inflammation and the disease state (36, 37). The observed underlying inflammation in the pathogenesis of BPS is seemingly orchestrated by pro-inflammatory cytokines and chemokines. Interleukin 6 (IL-6) secreted by immune cells is known to promote proliferation of prostatic cells by activation of JAK, STAT-3 and MAPK pathways and subsequently via androgen receptor induction (38). In experimental animal models of BPH, IL-6 levels have been reported to be elevated in the prostate (39). Our findings demonstrated increased expression of *Il6* mRNA in the prostate tissue as well. Lowered expression of *Il6* mRNA by Prostogrit suggests that it possesses the potential to address the chronic inflammation associated with the disease. This outcome may in part also explain the observed functional outcomes of the current study.

Prostogrit is comprised of the extracts of twenty-six botanical drugs. Further, it also contains fine powders obtained from the exudate of *Asphaltum punjabium* and the resin obtained from *Commiphora wightii*. In addition, it also contains two traditional mineral ingredients, which are classified as ‘Bhasmas’ in Ayurvedic system of medicine (**Table 1**). These constituents of Prostogrit have been traditionally used for prostatic enlargement. Phytochemical analysis of Prostogrit revealed the presence of eight phytometabolites, namely, gallic acid, 5-hydroxymethyl furfural (5-HMF), guggulsterone Z, guggulsterone E, benzoic acid, methyl gallate, cinnamic acid and piperine. Of these components, gallic acid is a natural compound reported to be present in many plants. It possesses antioxidant, anti-inflammatory and anti-microbial, properties (40). The extract of a Chinese medicinal plant, *Cynomorium songaricum* has been shown to demonstrate pharmacological activity in a rat model of BPH induced by administration of testosterone and estradiol (41). The observed *in vivo* efficacy of the extract has been attributed in part to the presence of gallic acid in the plant extract as the subsequent *in vitro* investigation of gallic acid in BPH-1 cell line demonstrated that the polyphenol could inhibit DHT or 17β-estradiol induced BPH-1 cell proliferation and modulated the steroid(s)-regulated androgen and estrogen receptor expressions (41). Although, not investigated in BPH, guggulsterones, the active principles of *Commiphora wightii* have demonstrated to promote apoptosis in prostate cancer cells and have been additionally reported to reduce the expression of androgen receptor (42). Piperine, also detected in Prostogrit is a bioactive compound with proven anti-proliferative, anti-inflammatory and antioxidant activities (43). It is hence conceivable that piperine might have contributed to the observed *in vivo* effects in the current experiment. Interestingly, piperine has also been reported to bind to the α_1A-_adrenoceptor, although the functional response of the observed binding has not been determined (44). Furthermore, the *in vivo* efficacy of *Juglans regia* extract in rat model of testosterone-induced BPH can be ascribed to the presence of methyl gallate, a polyphenolic compound and other phytometabolites like flavonoids and phenolic acids (45). Methyl gallate, also detected in Prostogrit is known to possess antioxidant, anti-inflammatory and anti-proliferative activities (46). Another phytometabolite present in Prostogrit is cinnamic acid. It is also the biologically active component of the traditional Chinese medicine Zi-Shen Pill, which has been reported to significantly reduce the prostate weight and prostatic index in rat model of testosterone-induced BPH and further ameliorate the histopathological changes in the prostate. The authors attributed the observed activity, in part to the presence of cinnamic acid in medicine (47).

Collectively, these cited scientific evidences suggest that the majority of phytometabolites detected in Prostogrit demonstrate biological activities both *in vitro* as well as in animal models of BPH. Accordingly, it is apparent that these compounds might have contributed to the observed pharmacological effects in the present study. Nevertheless, it is challenging to determine the phytometabolite which might have primarily contributed to the perceived pharmacological effects of Prostogrit. In our view, there is a high possibility that the detected biologically active phytometabolites may have rather functioned in synergistic manner.

Amongst the various botanical constituents present in Prostogrit, clinical evaluation of couple of these components in BPS patients, has been reported in public domain. Of these, an Ayurvedic medicine containing *Tribulus terrestris* as one of the components, demonstrated effectiveness in the initial management of BPS, by relieving both the static and dynamic components of the disease, when administered for 12-weeks to BPH patients (48). Furthermore, a *Solanum lycopersicum* var. *lycopersicum* (tomato) -based) food supplement exhibited clinical efficacy in symptomatic BPH patients, when administered for two months (49).

One of the recognized limitation of this preclinical study, is the absence of pharmacokinetic information. Existence of this data is important at arriving at a pharmacokinetic-pharmacodynamic correlation. Nevertheless, for medicines comprising of multiple botanical drugs, it is rather challenging to evaluate pharmacokinetics as opposed to single small molecule based pharmaceuticals. This difficulty is primarily due to the presence of complex, chemically diverse metabolites present in such formulations, and that too in slight amounts.

In conclusion, the present study has ascertained the *in vivo* pharmacodynamic effects of Prostogrit in moderating the principal characteristics of benign prostatic hyperplasia in rats, which was generated by the administration of TP+EB. The current study is the first report of the assessment of the *in vivo* efficacy of Prostogrit, in a clinically pertinent animal model of BPH, wherein the herbo-mineral medicine was administered prophylactically, to generate proof of concept information. On the basis of the results obtained in the present study, design of future studies is warranted, wherein the objective could be to assess the pharmacological effects of Prostogrit administered concurrently with disease induction and direct comparison of the pharmacological effects with finasteride, wherein a dose-response of finasteride can also be investigated parallelly. The future direction of Prostogrit research could be towards assessment of its non-clinical safety in rodents and non-rodents as per present regulatory guidelines. Additionally, these experiments also pave the way for detailed clinical investigation on human BPS patients under controlled clinical trial regimen, either as a standalone or as complementary therapy to the present standards of care.

## Data Availability

The original contributions presented in the study are included in the article/supplementary material. Further inquiries can be directed to the corresponding author.
